# ‘Sorrento’ and ‘Tulare’ Walnut Cultivars: Morphological Traits and Phytochemical Enhancement of Their Shell Waste

**DOI:** 10.3390/molecules29040805

**Published:** 2024-02-09

**Authors:** Elvira Ferrara, Danilo Cice, Simona Piccolella, Assunta Esposito, Milena Petriccione, Severina Pacifico

**Affiliations:** 1 Dipartimento di Scienze e Tecnologie Ambientali Biologiche e Farmaceutiche, Università degli Studi della Campania “Luigi Vanvitelli”, Via Vivaldi 43, 81100 Caserta, Italy; elvira.ferrara@unicampania.it (E.F.); simona.piccolella@unicampania.it (S.P.); assunta.esposito@unicampania.it (A.E.); severina.pacifico@unicampania.it (S.P.); 2 CREA-Centro di Ricerca Olivicoltura, Frutticoltura e Agrumicoltura, Via Torrino 3, 81100 Caserta, Italy; danilo93mhr@gmail.com

**Keywords:** *Juglans regia* L., walnut waste management, LC-HR MS/MS, polyphenols, tannins, antioxidant activity

## Abstract

Walnut processing generates considerable quantities of by-products that could be reprocessed into value-added products that have food and non-food applications. In this context, the aim of this study is to characterize the ‘Sorrento’ and ‘Tulare’ walnut cultivars using the UPOV guidelines and analyze the chemical composition and antioxidant activity of their shells. Insight into the chemical composition of the different granulometric fractions of walnut shell, obtained by sieving, was obtained following ultrasound-assisted extraction by Ultra-High-Performance Liquid Chromatography–High-Resolution Mass Spectrometry (UHPLC-HRMS). The total phenolic, flavonoid, and tannin content and antiradical capacity, obtained by DPPH and ABTS assays, and the Fe(III) reducing power of the extracts were also evaluated. The UHPLC-HRMS analysis indicated the presence of thirty-two compounds ascribable to four major classes of specialized metabolites. Furthermore, the extraction efficiency of gallic acid, ellagic acid derivatives, as well as glansreginin A, increased with the decrease in shell matrix particle size in contrast to chlorogenic acids and flavonoid glycosides. This is the first study to highlight new knowledge on the chemical composition of walnut shells. The results obtained demonstrate the feasibility of recovering valuable bioactive components from agro-waste that may be further valorized.

## 1. Introduction

Walnut (*Juglans regia* L.), also known as Persian walnut or English walnut, is an Old World tree belonging to the Juglandaceae family and one of the best-known and widely cultivated walnut species thanks to its good timber and high-quality nuts [1,2,3]. The origin and human-mediated expansion of walnut is still a matter of debate as its horticultural evolutionary history is characterized by a complex interaction of biogeographic and human forces [4,5]; however, it is believed that it probably originated in the Central Asian highlands [6,7,8] and from there spread to Europe as early as 1000 BC along ‘corridors’ such as the ‘Land Silk Road’ and ‘Persian Royal Road’ [9,10] with human migration and trade. Subsequently, the walnut has spread and adapted well to many temperate regions of the world, with several varieties cultivated mainly for the production of walnuts [2], whose nutritional value is influenced by the cultivar, the processing method, and by pedoclimatic conditions [11]. Walnut seeds (kernel) are known for their flavor and high nutritional value, serving as an excellent source of healthy monounsaturated and polyunsaturated fatty acids (omega-3 fatty acids) and a well-balanced omega-6/omega-3 ratio, as well as protein, fiber, and trace elements such as vitamins and minerals [12,13,14]. 

Their kernels are also an excellent source of phenolic acids, polyphenols, and other specialized metabolites [15,16,17] responsible for antioxidant and anti-inflammatory activity, which play an important role in protecting cells from oxidative damage [18] and pathologies [19,20,21].

Nowadays, several clinical and experimental studies have highlighted the potential health benefits deriving from the consumption of walnut kernels, including the reduction in pathologies related to oxidative stress, such as Alzheimer’s disease, cardiovascular risk, and diabetes [20,21,22,23,24], and they are a highly recommended food in the daily diet of humans [25]. Walnuts are marketed as a dry product and, before reaching the consumer’s table, undergo various post-harvest processes such as dehulling, drying, and shelling [26]. The walnut processing industry to produce shelled fruit gives rise to a large quantity of valuable by-products such as green husks, broken shells, and kernels, i.e., ‘waste’, that has enormous potential to be reused and valorized in various industrial sectors [27]. The green husk represents approximately 20% of the total walnut production, while the woody shell reaches almost 50% of the weight of the entire walnut [28,29], thus representing the largest portion of walnut by-products. In recent years, several studies have highlighted the properties of this bioresource both as a source of bioactive compounds [30,31,32] and as a biomaterial which, while usually burned, could be used, thanks to their polylobed sclereid cell tissues, in structures with other biopolymers for densification, as well as for impregnation and molding in a more sustainable bioeconomy [33,34]. In 2022/2023, global in-shell walnut production continues to increase, with China (about 1.4 tons) and the United States (700 tons) being the largest producers [35]. There is an increase in demand as two-thirds of exports are sold as ‘ready-to-use’ shelled products. In recent years, in fact, the demand for ready-to-use shelled walnuts has increased worldwide as a convenient, healthy, and nutritious snack [36].

Italy is the leading consumer of walnuts, but its cultivation has recorded a very strong decline in recent years despite the peninsula’s pedo-climatic conditions being suitable. Walnut cultivation is carried out in various Italian regions, but 30% of national production is concentrated in Campania (around 4500 tonnes) [37]. The local climate and environmental conditions in different cultivation areas have allowed for the diversification of different varieties adapt to specific agricultural ecosystems [38,39,40]. These genotypes, with particular bio-agronomic and organoleptic characteristics, constitute a significant reservoir of crop genetic diversity with good tolerance to abiotic and biotic stresses that can promote sustainable regional economic development [41]. Among these, the ‘Sorrento walnut’ is an indigenous Italian walnut originating from the Sorrento peninsula; today, it is cultivated throughout the Campania region (southern Italy), having become a mixture of heterogeneous genetic entities in terms of fruit size and yield, and marketed as ‘Sorrento walnut’ [42]. It is included in the list of typical and traditional products of the Campania Region [43]. Numerous studies have demonstrated wide genetic variability among ‘Sorrento’ genotypes using simple sequence repeat markers [38,42] but few works focus on compositional and morphological characterization [44], and to our knowledge, no data regarding the characteristics of waste products such as the shells are yet available. However, over the last 20 years, modern walnut orchards have been established using cultivars of great commercial interest obtained from Californian breeding programs by implementing innovative approaches to their management [45], while the cultivation of the Sorrento variety has been limited to traditional areas and systems. Among the cultivars of commercial interest, ‘Tulare’ is a walnut cultivar selected at the University of California (UC Davis) from a controlled crossing (Tehama × Serr) with good production and excellent yield used to create modern orchards in Italy. In this context, the aim of this study is to evaluate and compare both above-mentioned cultivars through different approaches. The phenological and morphological character traits were evaluated to provide a series of indications and information to distinguish the Sorrento and Tulare cultivars. Furthermore, since the walnut-processing industry gives rise to a large quantity of valuable by-products during the production of shelled fruit, phytochemical studies on the different components of the shell of the two varieties were carried out in order to outline new directions of use for local producers. For this purpose, samples of both varieties were collected and shelled. The obtained shells, further separated into different components, were subjected to differential pulverization procedures, and extracted by ultrasound-accelerated maceration using ethanol. In each component of the shell, the total content of condensed tannins, the total content of flavonoids and phenols, as well as the anti-radical capacity was evaluated. Furthermore, the relevant metabolic profile was recorded using untargeted UHPLC-HRMS (Ultra-High-Performance Liquid Chromatography–High-Resolution Mass Spectrometry).

## 2. Results and Discussion

### 2.1. Morphological and Phenological Traits

The thirty descriptors of the morphological and phenological characteristics of the walnut cultivars ‘Tulare’ and ‘Sorrento’ are reported in Table S1 (Supplementary Materials). The distinctness between the two cultivars was highlighted by the following parameters: vigor, habit, shape and surface of the fruit, shell valve closure, thickness, and yield. For each cultivar the description of the morphological and phenological characteristics is reported below.

‘Sorrento’ is a cultivar of high vigor, erect habit, apical fruiting, and intermediate male and female flowering. The bud shape is flabellate, while the leaf shape is medium elliptical. There are 1–2 female flowers per cluster, and the infructescence type is binate. The nut shape is elliptical oblong, and the shell has a smooth surface, imperfect closure, high thickness, light-brown color, moderately grooved surface texture, and medium size and yield. The color of the kernel of the endopleura is yellowish white, and the ease of removal from the shell is high. Harvesting takes place in the first ten days of October, and the fruits are suitable for both fresh and dry consumption due to their excellent organoleptic characteristics. 

‘Tulare’, on the other hand, is a cultivar with medium vigor, open habit, lateral fruiting on annual branches, and intermediate male and female flowering. The bud shape is flabellate, and leaf shape is medium elliptical. Additionally, in this case, there are 1–2 female flowers per cluster, and the infructescence type is binate. The nut shape is round, and the shell has a rough surface, perfect valve closure, medium thickness, light-brown color, moderately grooved surface texture, and large size and high yield. The kernel color of the endopleura is yellowish white, and it is easy to remove the shell. As with the ‘Sorrento’ cultivar, the nuts of the ‘Tulare’ cultivar are harvested in the first ten days of October, and the fruits with excellent organoleptic and nutritional features are consumed fresh or dried. Furthermore, ‘Tulare’ is characterized by fruiting on the lateral branches with higher productivity than ‘Sorrento’, which shows only terminal fruits.

**Figure 1:**
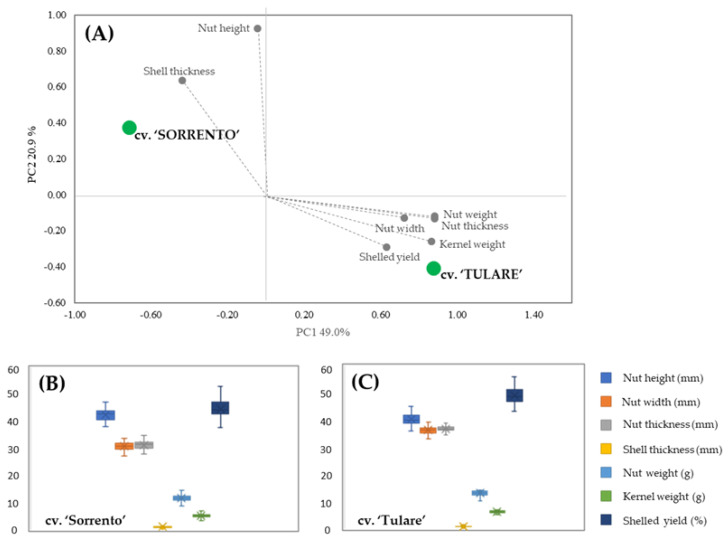
Morphometric analysis of ‘Sorrento’ and ‘Tulare’ walnut cultivars: PCA (**A**) and Box plot of ‘Sorrento’ (**B**) and ‘Tulare’ (**C**) of measured parameters. Student’s *t*-test revealed no significant differences among morphological traits of the two cultivars.

PCA analysis allowed for the discrimination between the two cultivars in terms of their carpological features (Figure 1A). The score plot showed two orthogonal PCs, accounting for 69.9% of the variance among the data. A marked segregation between the cultivars was highlighted, with ‘Sorrento’ located in the upper-left positive quadrant characterized by the shell thickness and nut height vectors, while ‘Tulare’ is in the lower-right negative quadrant with the vectors of the others measured parameters. The carpological characters of both cultivars are shown in the box plots (Figure 1B,C). Differences in nut height were revealed in both cultivars compared to fruit width and thickness, which showed higher values in ‘Tulare’ but lower endocarp thickness. The average nut weight is 11.82 ± 1.17 g and 13.24 ± 0.97 g, while the kernel weight is 5.27 ± 0.72 g and 6.38 ± 0.47 g for ‘Sorrento’ and ‘Tulare’, respectively. Our results are in agreement with Romano et al. [44] and McGranahan et al. [46] who highlighted an intervarietal difference in the total weight of the nuts ranging from 10.90 to 14.61 g and from 11.2 to 14.8 g, and in the kernel weight from 4.90 to 7.34 g and from 5.5 to 8.0 g in ‘Sorrento’ and ‘Tulare’, respectively. 

### 2.2. Extraction of Walnut Endocarp and Bioactive Compounds

In the past, the recovery of bioactive compounds from agricultural waste was carried out using conventional techniques that required long extraction times and a large amount of organic solvents but would damage to heat-sensitive compounds [47]. Nowadays, new eco-friendly and sustainable techniques have been proposed for the extraction of bioactive compounds with beneficial health effects, leading to an improvement in the yield and chemical compositional profile of the produced extracts [48,49]. Herein, ultrasound-assisted extraction (UAE) was applied to reduce extraction times, lower solvent consumption, and improve extraction rates and extract composition. Several studies have shown that optimizing extraction conditions during UAE results in extracts with a high yield of bioactive compounds while preserving their biological characteristics [50,51,52]. 

Particle size is one of the parameters that influences the efficiency of UAE. In several studies, particle size reduction by grinding agricultural waste samples improved the extraction efficiency due to increased solvent penetration and solute diffusion [30,48,51,53].

**Figure 2:**
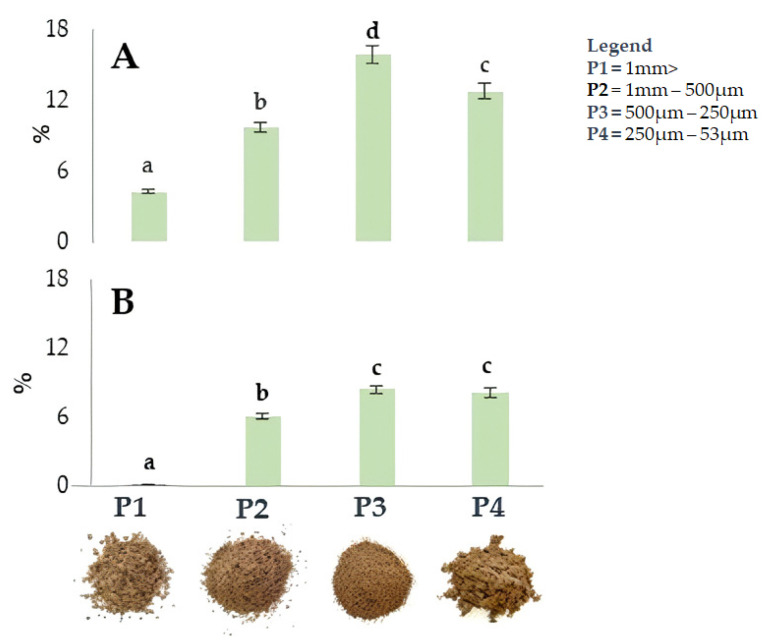
Yield (%) of ultrasound-assisted extraction in the four fractions, P1-P4, from ‘Tulare’ (**A**) and ‘Sorrento’ (**B**) walnut shell pulverized using different methods. Data are the mean ± SD of three independent measurements. Different letters denote a significant difference at the level of *p* < 0.05 using Duncan’s test.

Figure 2 shows the impact of particle size on extraction efficiency in four walnut shell fractions resulting from differential sieving based on particle size. Sieving was applied to the shells of both cultivars. The fraction with smaller particle sizes (P3 and P4) showed the highest extraction yield, reaching 15.9% (500–250 µm) and 12,7% (250–53 µm) in ‘Tulare’, while lower values of 8.4% (500–250 µm) and 8.2% (250–53 µm) were detected in the ‘Sorrento’ cultivar. Our hypothesis is that the different extraction efficiency values between the two walnut cultivars can be attributed to a tissue variation of the internal and external cellular layer structure of walnut shell. SEM observations on sections of California walnut shells highlighted that the outer layer is approximately 2.5–3 times thicker, denser, and harder than the inner layer, which is made up of loose and porous cells [30,54]. The latter are more easily penetrable after the rupture of the outer layer [30].

It is therefore expected that the differences in the yield extraction efficiency of the two analyzed cultivars can be attributed to the different thickness ratio between the two internal and external layers of the shell, and the fact that in Sorrento, the external layer is thicker than that of Tulare.

**Figure 3:**
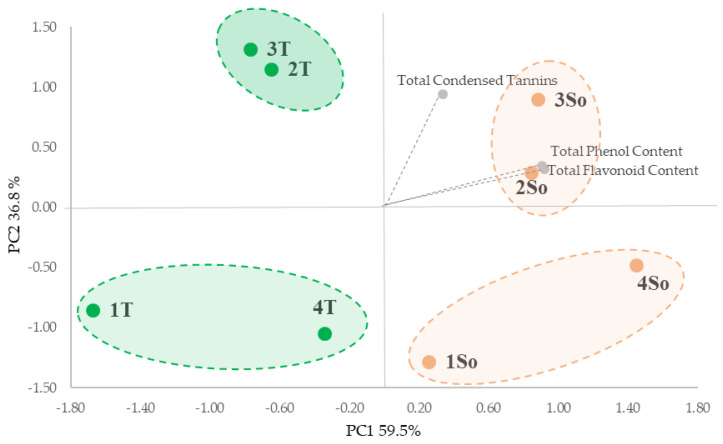
PCA of the walnut shell fractions from differential sieving based on particle size (as reported in Figure 2) and total bioactive content of the ‘Tulare’ (T; green) and ‘Sorrento’ (So; orange) cultivars.

All extracts were characterized in terms of total phenols (TPC), total flavonoids (TFC) and total condensed tannins (TCTs). The PCA analysis of the entire data set highlighted significant differences in the bioactive compound content based on shell particle size from ‘Sorrento’ and ‘Tulare’ cultivars (Figure 3). The PCA took into account the first two PCA axes with a cumulative variance of 96.30% (Figure 3) which allowed us to show the main segregation along the first axis (59.5% of variance), as determined by the total phenols content (TPC) and total flavonoids content (TFC) vectors, with the four fractions of the ‘Tulare’ cultivar in the negative square and the ‘Sorrento’ cultivar in the positive one. Furthermore the PC2 axis accounted for 36.8% of the variance, which evidenced the separation of granulometric fractions 2 and 3 from 1 and 4 for both cultivars on the basis of total tannins content (TCT). 

This is in agreement with the data reported by Han et al. [30] that highlighted the positive correlation between particle size and phenolic content, with smaller particle sizes significantly increased in relation to the surface area during extraction. Furthermore, we hypothesize that the greater content of bioactive compounds found in the ‘Sorrento’ cultivar could be linked to the outer layer of the shell which is probably thicker and denser and therefore able to store more phenolic compounds in dense structures containing high lignin and cellulose.

**Figure 4:**
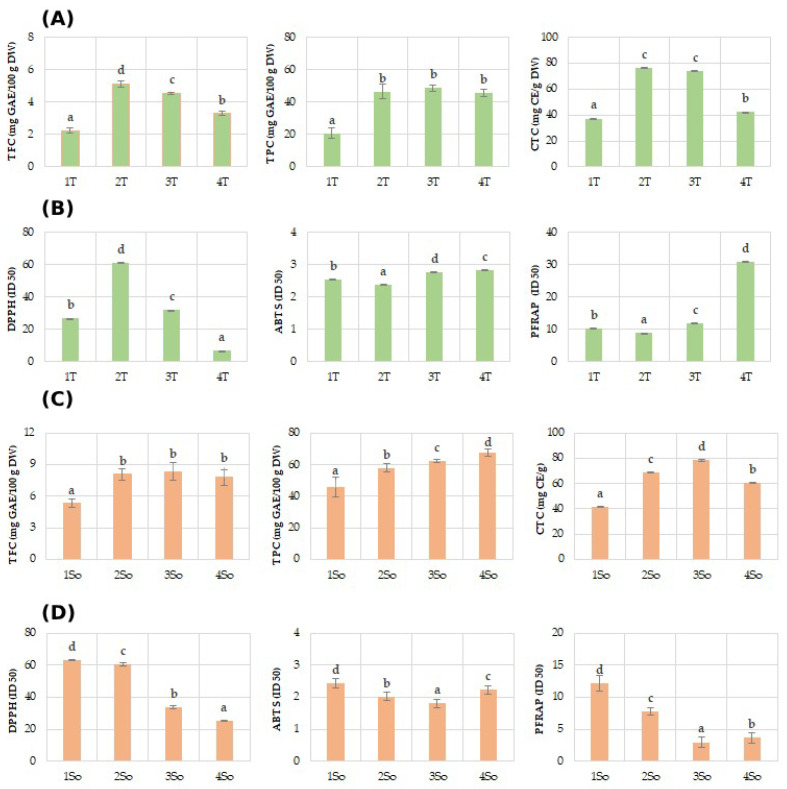
Phenols (TPC), flavonoids (TFC), and condensed tannins (TCT), and antioxidant assays (DPPH, ABTS, and PFRAP) evaluated in ‘Tulare’ (**A**,**B**) and ‘Sorrento’ (**C**,**D**) particle fractions. Data of the analyzed shell fractions are ordered according to the first axis gradient of PCA (Figure 3) for ‘Tulare’ and ‘Sorrento’. Data are the mean ± SD of three independent measurements. Different letters denote significant difference at the level of *p* < 0.05 by Duncan test.

Regarding the polyphenol content (TPC) of the shell fractions, they ranged between 28.68 mg GAE/100 g DW in 1T and 48.60 mg GAE/100 g DW in T3 of the Tulare cultivar (Figure 4A), while it showed higher values for the Sorrento (Figure 4C) cultivar, ranging between 45.88 mg GAE/100 g DW in 1S and 67.49 mg GAE/100 g DW. These results are in agreement with a recent study which highlighted a value of 52.8 mg GAEs/g DW attained using a shell particle size between 350 and 150 µm, an ultrasonic probe method, and a mixture of 50% ethanol/water as a solvent [30]. 

Similarly lower values of TFC were obtained for the ‘Tulare’ compared to the ‘Sorrento’ cultivar. In particular, shell fractions values of ‘Tulare’ ranged between 2.23 mg GAE/100 g DW in 1T and 5.10 mg GAE/100 g DW in T2 (Figure 4A), while it attained higher values for the ‘Sorrento’ cultivar, ranging between 5.31 mg GAE/100 g DW in 1S and 8.34 mg GAE/100 g DW in S3 (Figure 4C). The literature data on the flavonoid content are not available for the shell granulometric fractions, but the total flavonoid content values for the whole walnut shell have been reported to change from 4.86 to 80.40–162.5 mg/g of DW and of the extract, respectively [31]. The total condensed tannin, on the contrary, showed almost similar values in the two cultivars studied and for the analyzed fractions. In fact, shell fraction values of Tulare ranged between 37.23 mg GAE/100 g DW in 1T and 76.23 mg GAE/100 g DW in T2 (Figure 4A), while the values for Sorrento cultivar range between 41.59 mg GAE/100 g DW in 1S and 78.37 mg GAE/100 g DW in S3 (Figure 4C). Very few data are reported for condensed tannins in walnut shells. Queiros et al. [54] reported that condensed tannins accounted for only 60.1 mg CE/g of extract, using an extract obtained via ethanol/water (50/50, *v*/*v*) and an ultrasonic bath.

In this framework, it was observed that the extracts from walnut shells were able to increase the anti-radical and Fe(III) reducing efficacies of both studied cultivars, although with different levels in relation to the granulometric size fractions (Figure 4B,D). The results from the ferricyanide FRAP assay revealed that all the extracts, except to T4 for ‘Tulare’ and So1 for ‘Sorrento’, possessed compounds capable of donating a single electron to ferric ions. The antioxidant properties of walnut shell were determined by DPPH assay, with values ranging from 7.19 to 81.03 in relation to extraction method and analysis [27,31], while no literature data on ABTS and FRAP assays performed on the shells have been reported.

### 2.3. UHPLC-HR-MS/MS Analysis 

The extracts obtained by differential sieving from the shells of the cv. ‘Sorrento’ and ‘Tulare’ were analyzed for their polyphenolic profile using ultra-high-performance liquid chromatography coupled with high-resolution mass spectrometry. The identification of specialized metabolites was specifically achieved through a careful study of the TOF-MS/MS spectra, which allowed us to define the characteristic fragmentation patterns of each compound, and of the data in the literature. Thirty-two compounds were tentatively identified and are listed in Table 1. 

The compounds, which were not equally present in the extracts from the two investigated cultivars, were differentiable in four major classes of specialized metabolites. In fact, hydrolyzable tannins, together with gallic acid and ellagic acid (EA), and their glycosyl derivatives (hereafter all referred to as HTs) were distinguishable from the poorly represented condensed tannins (CTs), chlorogenic acids (CQAs), and flavonoid glycosides (FGs). Recently, transcriptomic and metabolic analyses highlighted that the peel of mature walnut fruits largely accumulates phenol compounds, mainly HTs [55], while no data are available for their content in the hard shell. Briefly, HTs in in the shells of the ‘Sorrento’ and ‘Tulare’ cvs consisted of fifteen compounds. Beyond gallic acid (**4**) and glucogallin (**3**) (Figure S1), whose content strongly depends on the overexpression of four UDP-glucosyltransferases in walnut [55], compounds **1** and **5** were hexahydroxydiphenic acid (HHDP) hexoside isomers. These compounds eluted at different retention times, while showing a similar TOF-MS/MS spectrum, with the ion at *m*/*z* 301.0 as the base peak attributable to the deprotonated ellagic acid (Figure S2). Although the ‘Sorrento’ cv consisted mostly of these compounds, and generally of HTs, HHDP-hexoside isomer 1 (**1**) showed, in both cultivars, a relative quantitative increase from P1 to P4, underlining its improved extraction efficiency with a decrease in the size of the matrix particles. Four *bis*-HHDP hexose isomers (compounds **2**, **7**, **9,** and **13**) with a deprotonated molecular ion at *m*/*z* 783.07 were also detected (Figure S3). The TOF-MS/MS of compound **2** broadly differed from that of the other isomers, which shared the neutral loss of ellagic acid, achieving an ion at *m*/*z* 481.06 (HHDP hexose). These compounds were likely pedunculagin or casuariin isomers, which were found as representative HHDP derivatives in walnuts [56]. The digalloyl hexose isomers (**6** and **10**), previously reported as walnut kernel and septum constituents, showed an deprotonated molecular ion at *m*/*z* 483.08, which underwent 152 Da neutral loss (*nl*), resulting in an ion at *m*/*z* 331.07 and/or 314 da *nl,* thereby achieving a gallate ion at *m*/*z* 169.01 [57] (Figure S4). Galloyl HHDP hexose isomers (**8**, **12,** and **20**), with an [M-H]^−^ ion at *m*/*z* 633.07, was previously determined to be the main compounds of dried walnut kernels [58], differing in the relative intensity of the ion at *m*/*z* 301.0, which was the base peak only in the TOF-MS/MS spectrum of compound **12** (Figure S5). The 302 Da *nl* was observable in the TOF-MS/MS spectra of compounds **18** and **22**, which are likely digalloyl-HHDP-hexose isomers, so much so that the shared [M-H]^−^ ion at *m*/*z* 785.09 furnished the ion at *m*/*z* 483.08 (Figure S6). Indeed, the deprotonated molecular ion lost the more favorable digalloyl hexose moiety, resulting in the ion at *m*/*z* 301.0. 

Finally, ellagic acid glycosides, previously found in the husk and pellicle of walnut [59], were detected. In particular, the deprotonated molecular ion at *m*/*z* 463.0498, which dissociated in its relative TOF-MS/MS spectrum, thus resulting in deprotonated ellagic acid, was in accordance with ellagic acid hexoside (**23**), while compound **24** was likely an ellagic acid pentosyl hexoside (Figure S7). In fact, the deprotonated molecular ion underwent neutral losses of 132 Da and 162 Da, achieving the ions at *m*/*z* 463.05 and 433.04, respectively. Two ellagic acid pentoside isomers (**28** and **29**) were also tentatively identified, mainly in the ‘Tulare’ cv., while ellagic acid (**30**) was found abundant in the ‘Sorrento’ cv (Figure S8). 

Despite the evidence that walnut is a rich source of proantocyanidins, only two isomers (**16** and **17**) were tentatively identified in the investigated extracts, and their TOF-MS/MS spectra were in accordance with procyanidins B2 (Figure S9). 

‘Tulare’ cv. extracts were rich in chlorogenic acids and flavonoid glycosides. Indeed, among the identified chlorogenic acids (compounds **11**, **14**, **15**, **19,** and **21**), ‘Tulare’ cv. extracts mostly contained 3-*O*-caffeoyl quinic acid (**11**) and 3-*O*-*p*-coumaroyl quinic acid (**15**). The latter was present in more than 69% of the CQAs constituents, with the sole exception of the P4 ‘Tulare’ sample, while 4-*O*-caffeoyl quinic acid (**19**) was the third and less abundant CQA among the ‘Tulare’ extracts [60,61]. The ‘Sorrento’ cv., despite boasting a lower CQA content, also contained 5-*O*- (**14**) and 4-*O*-*p*-coumaroyl quinic acid (**21**) isomers. All these compounds were surprisingly extracted with the highest efficiency from P1 shells, while they are reported as constituents of green husks, leaves, and male flowers. The TOF-MS/MS spectra of the chlorogenic acids in walnut shells are shown in Figure S10.

**Figure 5:**
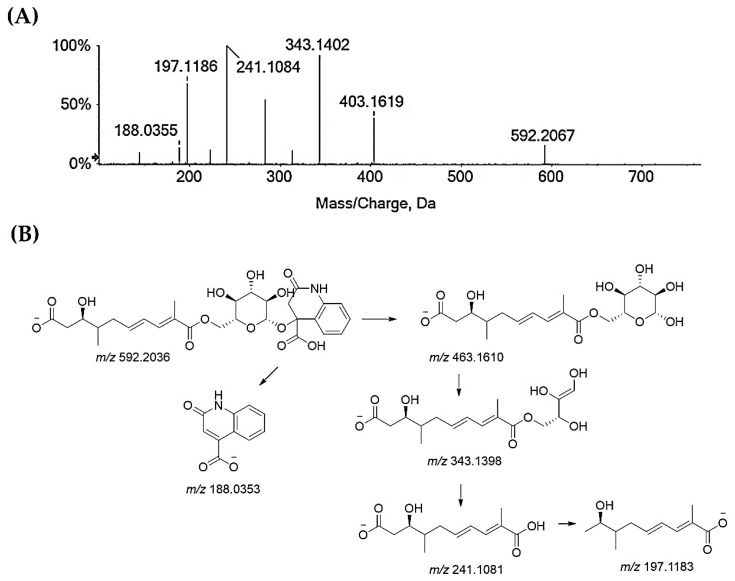
(**A**) TOF-MS/MS spectrum of compound **31**, which is likely glansreginin A, and (**B**) hypothesized fragmentation pattern. Theoretical *m*/*z* values are below each structure.

Compounds **25–27** and **32** are flavonoids. Compounds **25** and **26** are taxifolin pentoside isomers (Figure S11). Recently, two isomers of taxifolin-3-*O*-arabinofuranoside were isolated [62]. Taxifolin xyloside was also reported as being a constituent of diaphragma juglandis fructus, which is the dried woody septum inside the walnut hull. This fruit part is reported to be rich in flavonoids [63]. Compound **27** is quercetin galloyl hexoside. In fact, the deprotonated molecular ion lost 152 Da, resulting in an quercetin hexoside anion at *m*/*z* 463.0877, which, in turn, provided the deprotonated quercetin aglycone at *m*/*z* 301.03 after losing 162 Da (Figure S12). Quercetin-3-*O*-(6″-*O*-galloyl)-β-d-galactopyranoside was recently isolated from an antidiabetic extract of Diaphragma juglandis Fructus, which is able to improve diabetes symptoms via the AKT/FoxO1 signaling pathway [64]. Lastly, the TOF-MS/MS spectra of compound **32** agreed with a quercetin deoxyhexoside, likely the quercetin rhamnoside (Figure S13), which was previously found in green walnut liqueur [65] and walnut leaves [66]. Beyond these major classes of specialized metabolites, glansreginin A (**31**), a dicarboxylic acid derivative, ([M-H]^−^ ion at *m*/*z* 592.2036) was tentatively identified. The deprotonated molecular ion fragmented to give the ion at *m*/*z* 463.16 following the neutral loss of the 2-oxo-1,2-dihydroquinoline-4-carboxylic acid residue, which was also detectable in the form of its anion at *m*/*z* 188.03 (Figure 5). The cross-ring cleavage of the sugar moiety provided the ion at *m*/*z* 343.14, while the ion at *m*/*z* 241.10 was in accordance with 9-carboxy-3-hydroxy-4-methyldeca-6,8-dienoate, which underwent carbon dioxide loss to form the ion at *m*/*z* 197.12. Glansreginin A is an indicator of the quality of walnuts (*Juglans* spp.), and it was shown to exert antibacterial activity towards Gram-positive bacteria, such as *Staphylococcus aureus* and *Bacillus anthracis* [67], and to be able to counteract inflammation, even if it does not exhibit antioxidant potential [68]. The content of glansreginin A varied among walnut cultivars, so much so that it was found to range from 6.8 mg/kg to 47.0 mg/kg [64]. 

**Figure 6:**
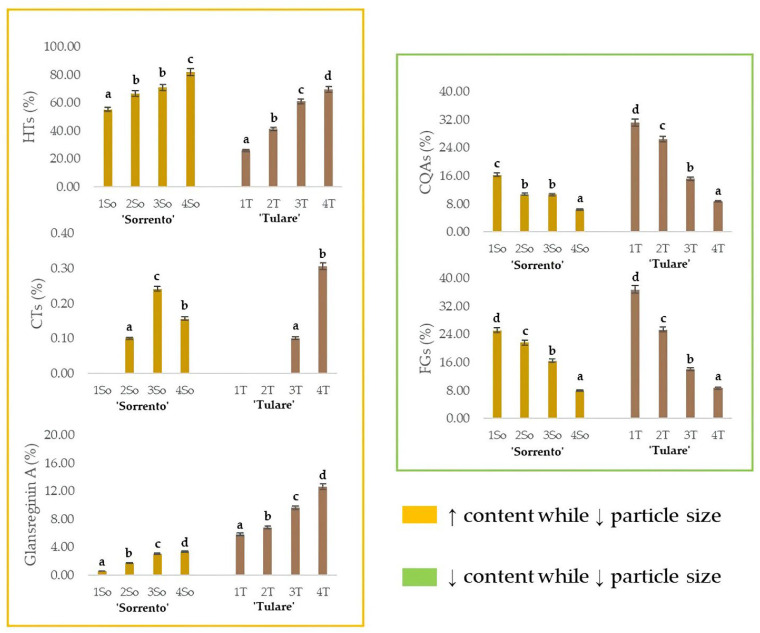
Relative quantitation of the different classes of specialized metabolites in ‘Sorrento’ and ‘Tulare’ extracts. HTs = Hydrolyzable tannins, plus gallic acid, ellagic acid, and their glycosides; CTs = Condensed tannins; CQAs = chlorogenic acids; FGs = flavonoid glycosides. The relative content of glansreginin A is also depicted. Data are the mean ± SD of three independent measurements. Different letters denote significant difference at the level of *p* < 0.05 using Duncan’s test.

The relative content of glansreginin A, as well as of the HTs, CTs, CQAs and FGs, in the ‘Sorrento’ and ‘Tulare’ extracts, are graphed in Figure 6. As observed for the HTs, the extraction efficiency of the dicarboxylic acid derivative augmented while decreasing the particle size of the shell matrix. These findings are in line with the optimization of preferential extraction procedures for obtaining compounds attributable to specific metabolic classes of interest. It is especially worthy of note that compounds commonly identifiable in the edible components of the walnut, or in waste parts that are less difficult to process, can be isolated from this residue, which, as already mentioned previously, is the one with the greatest impact. The antioxidant, anti-inflammatory, antiatherosclerotic, analgesic, cholesterol-absorption-reducing, and antibacterial properties of glansreginin A and polyphenols [69,70] make this residue a mine to explore. On the other hand, the lipid-lowering activity, attributed to the glansreginin A and ellagic acid mainly found in walnut flour extract [71], places the walnut shell as an invaluable nutraceutical resource.

## 3. Materials and Methods

### 3.1. Walnut Samples

**Figure 7:**
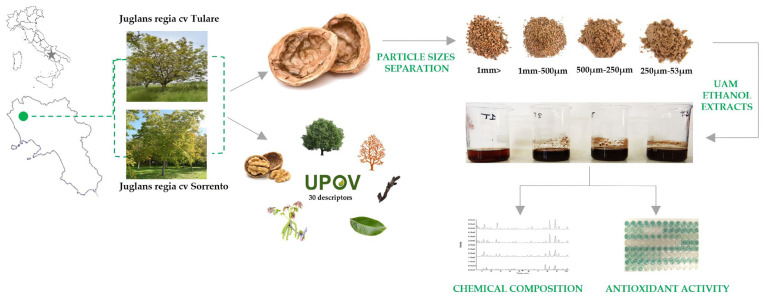
Sampling site of *Juglans regia* cvs ‘Sorrento’ and ‘Tulare’ and scheme of experimental procedure.

The fruits of *Juglans regia* cvs ‘Sorrento’ and ‘Tulare’ (*n* = 500) were randomly harvested in October 2022 in a commercial orchard located North of Caserta, southern Italy (41.157557 N; 14.147334 E) (Figure 7). Morphological and phenological traits of ‘Sorrento’ and ‘Tulare’ walnuts were detected using 50 trees per cultivar to test the distinctness, uniformity, and stability of walnut varieties using the Union for the Protection of New Plant Varieties (UPOV) guidelines [72]. Walnut fruits were shipped to the laboratory, checked for physical and biotic characteristics, and hand-shelled (*n* = 100) to separate the husk, shells and kernels. The shells were ground using a mill (Sorvall DuPont Omni Mixer, United States). The powder obtained from the shells was separated into four fractions with different particle sizes (53–250 µm, 250 µm–500 µm, 500 µm–1 mm, and >1 mm), using a vibrating sieving apparatus (Retsch, Haan, Germany). Each fraction obtained was weighed and stored for further analysis. Experimental procedures are reported in Figure 7.

### 3.2. Extraction of Sorrento and Tulare cv. Walnut Shell

Four shells fractions were extracted using the same method described by Ferrara et al. [48], and the extraction percent yield was determined. All obtained samples were stored at 4 °C until further analysis.

### 3.3. Determination of DPPH and ABTS Radical Scavenging Capacity

The scavenging ability of the alcoholic extracts obtained by the four shells’ particle size fractions were evaluated for 2,2-diphenyl-1-picrylhydrazyl acid (DPPH) and 2,2-azinobis-(3-ethylbenzothiazolin-6-sulfonic acid (ABTS). DPPH and ABTS assay were carried out according to Ferrara et al. [48]. The DPPH alcoholic solution (9.4 × 10^−5^ M) was added to shell walnut extracts in order to reach final concentration levels equal to 1, 5, 10, 50, 100, and 200 µg/mL. After stirring for 15 min, the absorbance was read at 515 nm using a Wallac Victor3 spectrophotometer (Perkin Elmer/Wallac; Waltham, MA, USA). The ABTS radical cation solution was prepared with an initial absorbance of 0.70 at 734 nm and added to shell walnut extracts to obtain the final tested concentrations (1, 5, 10, 50, 100, and 200 µg/mL). The absorbance was measured after 6 min of incubation.

For each sample and tested concentration, three technical replicates were created. A positive control was established by using Trolox (4, 8, 16, and 32 µM). The results were expressed as an ID_50_ value, which is the sample dose level required for a 50% scavenge rate using radical probes. 

### 3.4. Determination of Fe(III) Reducing Power

Shell walnut extracts (1, 5, 10, 50, 100, and 200 µg/mL final concentration levels) in a NaH_2_PO_4_/Na_2_HPO_4_ buffer (0.2 M, pH 6.0) were tested for their ferric ion reducing power using the PFRAP method, as reported in Formato et al. [73]. The results were expressed as an ID_50_ value, which is the sample concentration able to reduce Fe(III) ions by 50%.

### 3.5. Determination of Phenol and Flavonoid Content

The total phenol content (TPC) of shell walnut extracts was determined using microplates and the Folin–Ciocalteu method, using 1 mg and 0.5 mg of the extract in the assay solution, containing Na_2_CO_3_ (7.5% *w*/*v*) and Folin–Ciocalteu reagent (FCR), as described by Ferrara et al. [47]. The absorbance was read at 765 nm using a Wallac Victor3 spectrophotometer (Perkin Elmer/Wallac; Waltham, MA, USA). The results were expressed as gallic acid milligram equivalents (GAE) per 100 g of dry material. The assessment of the total flavonoid content (TFC) was performed using the aluminum chloride colorimetric method. Each shell walnut extract (1 and 2 mg) was dissolved in an aqueous NaNO_2_ solution (5%, *w*/*v*), and then an AlCl_3_ solution (10%, *w*/*v*) was added. An NaOH aqueous solution (1.0 M) was also added to the assay mixture, which was allowed to react for 6 min and then diluted with distillate water until a final volume of 10 mL was reached. The absorbance was read at 510 nm against the blank (water). The results were expressed as milligrams of catechin equivalents per 100 g of DW.

### 3.6. Determination of Condensed Tannins

Condensed tannins were recorded in shell walnut extracts according to Ferrara et al. [48]. The absorbance was read at 550 nm. The results were expressed in milligrams of catechin equivalent per milliliter (mg/mL).

### 3.7. UHPLC-ESI-QqTOF-MS/MS Analysis

Shell walnut samples (10 mg/mL) were analyzed by injecting a volume of 2.0 µL into the Shimadzu NEXERA UHPLC system (manufactured by Shimadzu, Tokyo, Japan), using the Luna^®^ Omega C18 column with a particle size of 1.6 µm and dimensions of 50 × 2.1 mm i.d., and eluent A (water + HCOOH 0.1%) and eluent B (acetonitrile + HCOOH 0.1%). The separation process involved implementing a gradient with an increasing % of eluent B over time: 0–3.00 min from 2% to 5% B; 3.00–5.00 min at 5% B, 5.00–8.00 min from 5% to 20% B; 8.00–10.00 min from 20% to 40% B; and 10.00–12.00 min from 40% to 98% B. The system was held for 1 min at 98% B, while the initial condition (2% B) was restored at 15.00 min. Column rebalancing was carried out at 17.00 min, maintaining a composition of 2% B. The flow rate was set at 0.5 mL/min. For the mass spectrometric (MS) analysis, a hybrid Q-TOF MS instrument was used, specifically the AB Sciex Triple TOF^®^ 4600. The instrument operated in negative ElectroSpray (ESI) mode, employing an APCI probe for automatic mass calibration using the Calibration Delivery System. The analysis included one full scan (TOF) in the mass range of 100–1500 Da, with a dwell time of 250 ms, as well as eight IDA MS/MS scans with a dwell time of 100 ms within the mass range of 80–1500 Da. Various parameters were set for optimal performance, including a curtain gas at 35 psi, nebulizer gas and heated gas both at 60 psi, ion spray voltage at 4500 V, ion source temperature at 600 °C, and declustering potential at −70 V. The collision energy was kept at −35 V. In addition, an ionic scattering (CES) of 15 V was used. The instrument was operated using Analyst^®^ TF 1.7 software (produced by AB Sciex, Concord, ON, Canada, 2016), while the processing of the acquired data was performed using PeakView^®^ version 2.2 software (produced by AB Sciex, Concord, ON, Canada, 2016).

### 3.8. Statistical Analysis

*Morphological traits*—Thirty morphological (n = 500 fruit) and phenological (n = 50 trees) traits descriptors were measured, and for each mean value obtained, a status and a number were assigned according to the UPOV guidelines. Box plots were created using Excel analysis, and principal component analysis (PCA) was used to describe the relationship between the morphometric data and to explore the similarity/dissimilarity of the studied walnut cultivars. T-student for mean comparisons were used (*p* < 0.05). PCA analysis was performed using the SPSS software package, version 20.0 (SPSS Inc., Chicago, IL, USA).

*Phytochemical analysis and bioactivity*—All data were reported as mean ± standard deviation (SD) and were by performing three replicate measurements for each sample. One-way ANOVA and the Duncan test for mean comparisons were used (*p* < 0.05) using the SPSS software package, version 20.0 (SPSS Inc., Chicago, IL, USA).

## 4. Conclusions

Walnut processing produces enormous amounts of walnut shells which could be used towards a sustainable recycling and reuse process to produce value-added products that have food and non-food applications, contributing to the shift from a linear to a circular economy. Herein, the comprehensive bio-agronomic characterization of two walnut cultivars, ‘Sorrento’ and ‘Tulare’, cultivated in the Campania Region (Italy) highlighted important intervarietal differences. Furthermore, the chemical approach used to characterize shell walnut highlighted that ultrasound-assisted maceration in ethanol is an eco-friendly and sustainable technique that allows for the recovery of several bioactive chemical compounds from walnut shells, adding new value to this agro-waste.

## Figures and Tables

**Table 1:** TOF-MS/MS data of compounds tentatively identified in investigated extracts from walnut shells. Ring Double Bond values (RDB) are listed. TOF-MS/MS spectra are provided in the Supplementary Materials.

Peak	RT (min)	Tentative Assignment	Formula	[M-H]^−^ Found (*m*/*z*)	Error (ppm)	RDB
**1**	0.432	Hexahydroxydiphenoyl-D- hexoside isomer 1	C_20_H_18_O_14_	481.0637	2.7	12
**2**	0.640	Bis-HHDP-hexose isomer 1	C_34_H_24_O_22_	783.0686	0.7	23
**3**	0.687	Glucogallin	C_13_H_16_O_10_	331.0671	1.3	6
**4**	0.750	Gallic acid	C_13_H_16_O_10_	169.0142	4.5	5
**5**	0.933	Hexahydroxydiphenoyl-D- hexoside isomer 2	C_20_H_18_O_14_	481.0624	3.4	12
**6**	1.00	Digalloyl hexose isomer 1	C_20_H_20_O_14_	483.0801	4.3	11
**7**	1.042	Bis-HHDP-hexose isomer 2	C_34_H_24_O_22_	783.0718	4.0	23
**8**	1.317	Galloyl-HHDP-hexose isomer 1	C_20_H_26_O_23_	633.0778	−2.2	8
**9**	1.517	Bis-HHDP-hexose isomer 3	C_34_H_24_O_22_	783.0728	5.3	23
**10**	1.892	Digalloyl hexose isomer 2	C_20_H_20_O_14_	483.0799	3.9	11
**11**	2.092	3-*O*-Caffeoylquinic acid	C_16_H_18_O_9_	353.0880	0.5	8
**12**	2.604	Galloyl-HHDP-hexose isomer 2	C_20_H_26_O_23_	633.0768	−3.8	8
**13**	2.619	Bis-HHDP-hexose isomer 4	C_34_H_24_O_22_	783.0710	3.0	23
**14**	2.866	Coumaroylquinic acid isomer 1	C_16_H_18_O_8_	337.0921	−2.3	8
**15**	2.952	Coumaroylquinic acid isomer 2	C_16_H_18_O_8_	337.0936	2.1	8
**16**	3.522	Procyanidin B2 isomer 1	C_30_H_26_O_12_	577.1368	2.9	18
**17**	3.745	Procyanidin B2 isomer 2	C_30_H_26_O_12_	577.1375	4.1	18
**18**	3.766	Digalloyl-HHDP-hexose isomer 1	C_27_H_30_O_27_	785.0902	−0.6	13
**19**	4.099	4-*O*-caffeoylquinic acid	C_16_H_18_O_9_	353.0889	2	8
**20**	4.831	Galloyl-HHDP-hexose isomer 3	C_20_H_26_O_23_	633.0764	−4.4	8
**21**	5.587	Coumaroylquinic acid isomer 3	C_16_H_18_O_8_	337.0915	−4.1	8
**22**	6.720	Digalloyl-HHDP-hexose isomer 2	C_27_H_30_O_27_	785.0876	−3.3	13
**23**	7.588	Ellagic acid hexoside	C_20_H_16_O_13_	463.0498	−4.4	13
**24**	7.611	Ellagic acid pentosyl hexoside	C_25_H_24_O_17_	595.0913	−4.7	14
**25**	7.640	Taxifolin−3-*O*-pentoside isomer 1	C_20_H_20_O_11_	435.0946	3.0	11
**26**	7.856	Taxifolin−3-*O*-pentoside isomer 2	C_20_H_20_O_11_	435.0933	−0.4	11
**27**	8.179	Quercetin galloyl hexoside	C_28_H_24_O_16_	615.0981	−1.7	17
**28**	8.192	Ellagic acid pentoside isomer 1	C_19_H_14_O_12_	433.0423	2.4	12
**29**	8.201	Ellagic acid pentoside isomer 2	C_19_H_14_O_12_	433.0429	3.8	12
**30**	8.272	Ellagic acid	C_14_H_6_O_8_	300.9990	3.0	12
**31**	8.804	Glansreginin A	C_28_H_35_NO_13_	592.2036	2.8	12
**32**	8.927	Quercetin-3-*O*-deoxyhexoside	C_21_H_20_O_11_	447.0933	2.3	12

## Data Availability

Data are contained within the article and Supplementary Materials.

## Supplementary Materials

The following supporting information can be downloaded at: https://www.mdpi.com/article/10.3390/molecules29040805/s1. Table S1: Morphological traits of *Juglans regia* cvs ‘Tulare’ and ‘Sorrento’ according to UPOV guidelines; Figure S1: TOF-MS/MS spectra of compounds **3** (A) and **4** (B), and chemical structures of their deprotonated molecular ions. The theoretical *m*/*z* value is below each structure; Figure S2: TOF-MS/MS spectra of compounds **1** (A) and **5** (B), which are likely hexahydroxydiphenic acid (HHDP) hexoside isomers; Figure S3: TOF-MS/MS spectra of compounds **2** (A), **7** (B), **9** (C), and **13** (D); Figure S4: TOF-MS/MS spectra of compounds **6** (A) and **10** (B); Figure S5: TOF-MS/MS spectra of galloyl HHDP hexose isomers: **8** (A), **12** (B), and **20** (C); Figure S6: TOF-MS/MS spectra of compounds **18** and **22**, likely digalloyl-HHDP-hexose isomers; Figure S7: TOF-MS/MS spectra of ellagic acid glycosides **23** (A) and **24** (B); Figure S8: TOF-MS/MS spectra of compounds **27** (A) and **28** (B), likely ellagic acid pentoside isomers (whose fragmentation pattern is showed below), and of compound **29** (C); Figure S9: TOF-MS/MS spectra of B-type procyanidin isomers **16** (A) and **17** (B); Figure S10: TOF-MS/MS spectra of chlorogenic acids: (A) 3-O-caffeoyl quinic acid (**11**); (B) 4-O-caffeoyl quinic acid (**19**); (C) 5-O-*p*-coumaroyl quinic acid (**14**); (B) 3-O-*p*-coumaroyl quinic acid (**19**); (E) 4-O-*p*-coumaroyl quinic acid (**21**); Figure S11: TOF-MS/MS spectra of taxifolin pentoside isomers: (A) **25**; (B) **26**; Figure S12: TOF-MS/MS spectra of quercetin glycoside **27**; **Figure S13.** TOF-MS/MS spectra of quercetin glycoside **32**.
