# Supplementary material for: ‘Sorrento’ and ‘Tulare’ Walnut Cultivars: Morphological Traits and Phytochemical Enhancement of Their Shell Waste
Source: Molecules. 2024 Feb 9;29(4):805. doi: 10.3390/molecules29040805 (PMC10893203; doi:10.3390/molecules29040805)
Supplement: Supplementary file 1 [file molecules-29-00805-s001.zip › molecules-2832759-supplementary.pdf]

Supplementary Materials

Tulare	DESCRIPTOR	ATTRIBUTED
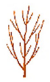	Tree: growth habit<br>Tree: branching	*Spreading*<br>*Medium*
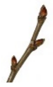	Bud: shape	*Flabellate*
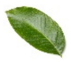	Leaflet: shape	*Medium elliptic*
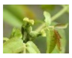	Plant: second flowering<br>Female flower: number per cluster<br>Female flower: intensity of yellow color of stigma<br>Infructescence: type	*Absent*<br>*1-2*<br>*Light*<br>*Binate*
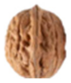	Nut: size<br>Nut: shape in ventral view<br>Nut: shape in lateral view<br>Nut: shape in cross section<br>Nut: shape of base in lateral view<br>Nut: shape of apex lateral view<br>Nut: length of tip<br>Nut: extent of pad around suture<br>Nut: prominence of pad on suture<br>Nut: width of pad on suture in lateral view<br>Nut: depth of groove along pad on suture<br>Nut: structure of surface of shell<br>Nut: color of shell<br>Nut: thickness of dividing membranes<br>Nut: inner pleat wall of shell<br>Nut: thickness of shell	*Medium*<br>*Broad elliptic*<br>*Broad elliptic*<br>*Oblate*<br>*Rounded*<br>*Obtuse*<br>*Long*<br>*On whole length*<br>*Strong*<br>*Medium*<br>*Shallow*<br>*Moderately grooved*<br>*Light brown*<br>*Very thin*<br>*Papery*<br>*Medium*
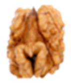	Kernel: color of endopleura<br>Kernel: percentage of weight relative to total weight of nut<br>Kernel: ease of removal from shell	*Yellowish white*<br>*Medium*<br>*Easy*
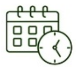	Time of beginning of female flowering<br>Time of male flowering compared to female flowering<br>Time of harvest maturity	*Medium*<br>*Before*<br>*Medium*

	Sorrento DESCRIPTOR	ATTRIBUTED
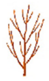	Tree: growth habit	***Upright***
	Tree: branching	***Medium***
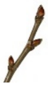	Bud: shape	***Flabellate***
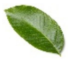	Leaflet: shape	***Medium elliptic***
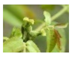	Plant: second flowering	***Absent***
	Female flower: number per cluster	***1-2***
	Female flower: intensity of yellow color of stigma	***Light***
	Infructescence: type	***Binate***
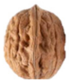	Nut: size	***Medium***
	Nut: shape in ventral view	***Oblong***
	Nut: shape in lateral view	***Ovate***
	Nut: shape in cross section	***Oblate***
	Nut: shape of base in lateral view	***Truncate***
	Nut: shape of apex lateral view	***Obtuse***
	Nut: length of tip	***Medium***
	Nut: extent of pad around suture	***On upper 2/3***
	Nut: prominence of pad on suture	***Strong***
	Nut: width of pad on suture in lateral view	***Broad***
	Nut: depth of groove along pad on suture	***Deep***
	Nut: structure of surface of shell	***Moderately grooved***
	Nut: color of shell	***Light brown***
	Nut: thickness of dividing membranes	***Very thin***
	Nut: inner pleat wall of shell	***Ligneous***
	Nut: thickness of shell	***Thick***
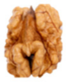	Kernel: color of endopleura	***Yellowish brown***
	Kernel: percentage of weight relative to total weight of nut	***Medium***
	Kernel: ease of removal from shell	***Very easy***
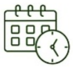	Time of beginning of female flowering	***Medium***
	Time of male flowering compared to female flowering	***Before***
	Time of harvest maturity	***Medium***

Table S1. *Juglans regia* cvs 'Tulare' and 'Sorrento' morphological traits according to UPOV guidelines.

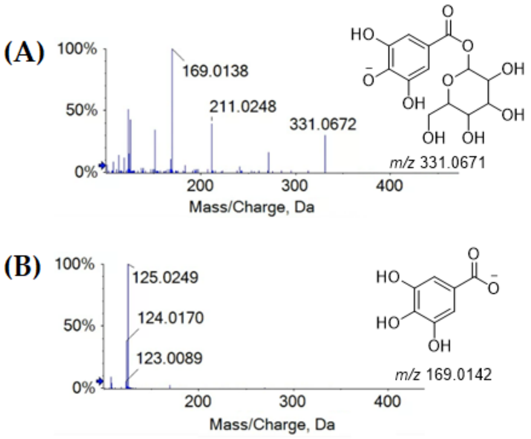

Figure S1. TOF-MS/MS spectra of compounds **3** (A) and **4** (B), and chemical structures of their deprotonated molecular ions. The theoretical *m/z* value is below each structure.

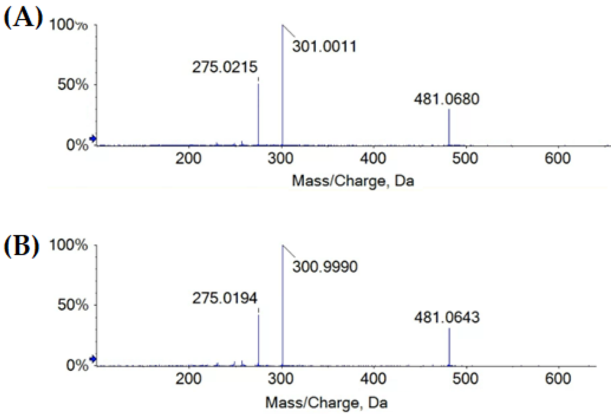

Figure S2. TOF-MS/MS spectra of compounds **1** (A) and **5** (B), which were likely hexahydroxydiphenic acid (HHDP) hexoside isomers.

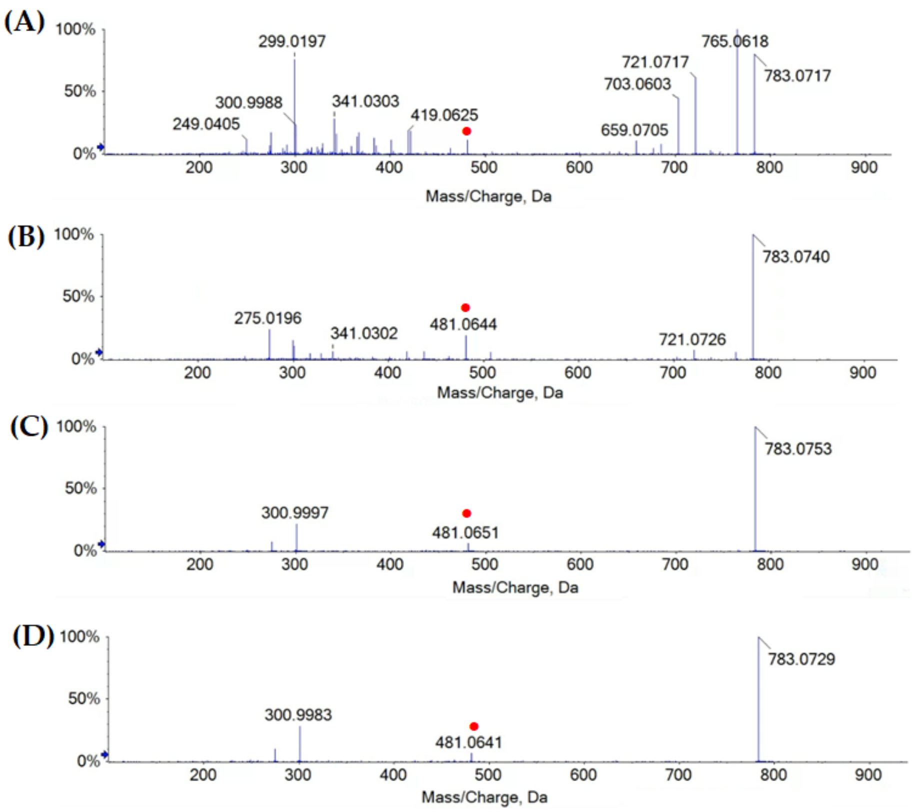

Figure S3. TOF-MS/MS spectra of compounds **2** (A), **7** (B), **9** (C), and **13** (D)

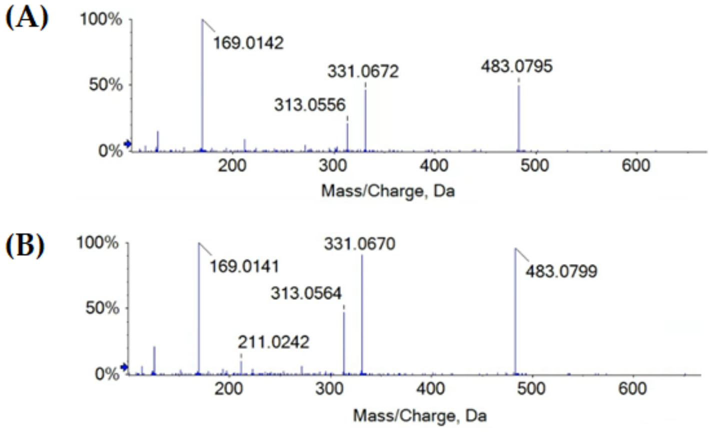

Figure S4. TOF-MS/MS spectra of compounds **6** (A), and **10** (B)

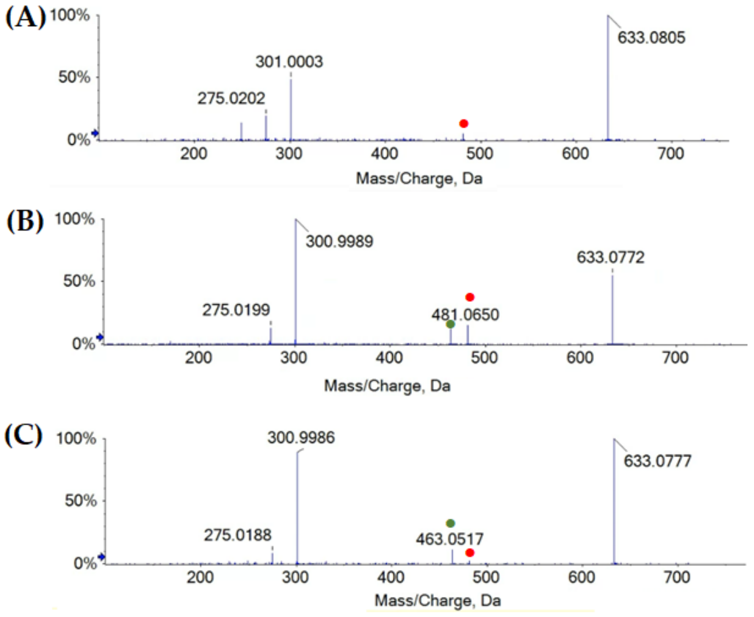

Figure S5. TOF-MS/MS spectra of galloyl HHDP hexose isomers: **8** (A), **12** (B), and **20** (C).

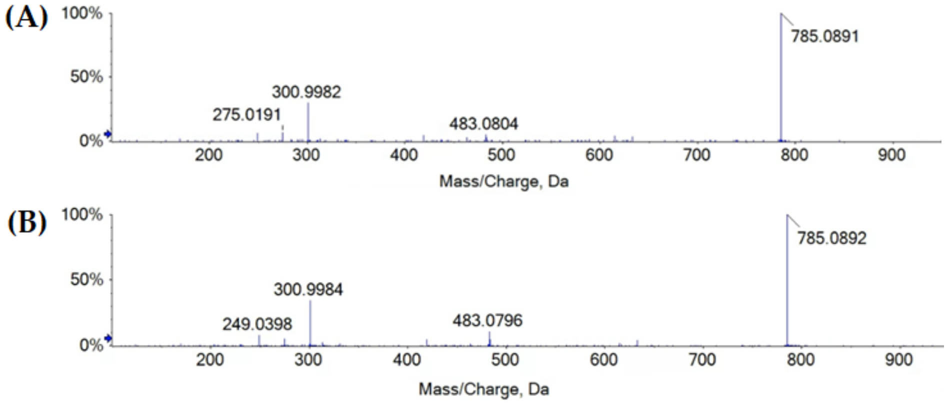

Figure S6. TOF-MS/MS spectra of compounds **18** and **22**, likely digalloyl-HHDP-hexose isomers.

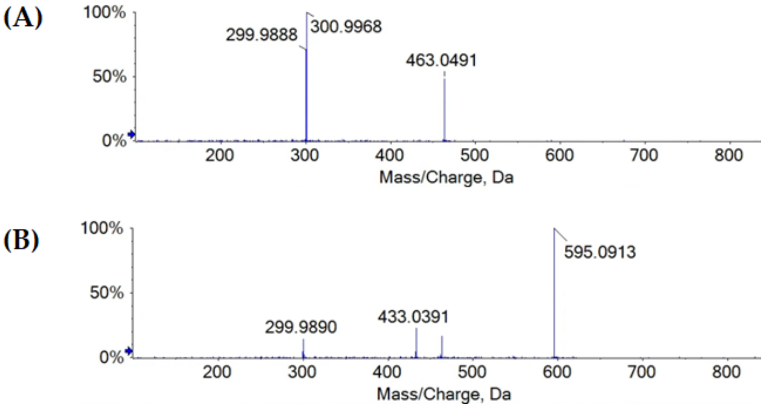

Figure S7. TOF-MS/MS spectra of ellagic acid glycosides **23** (A) and **24** (B).

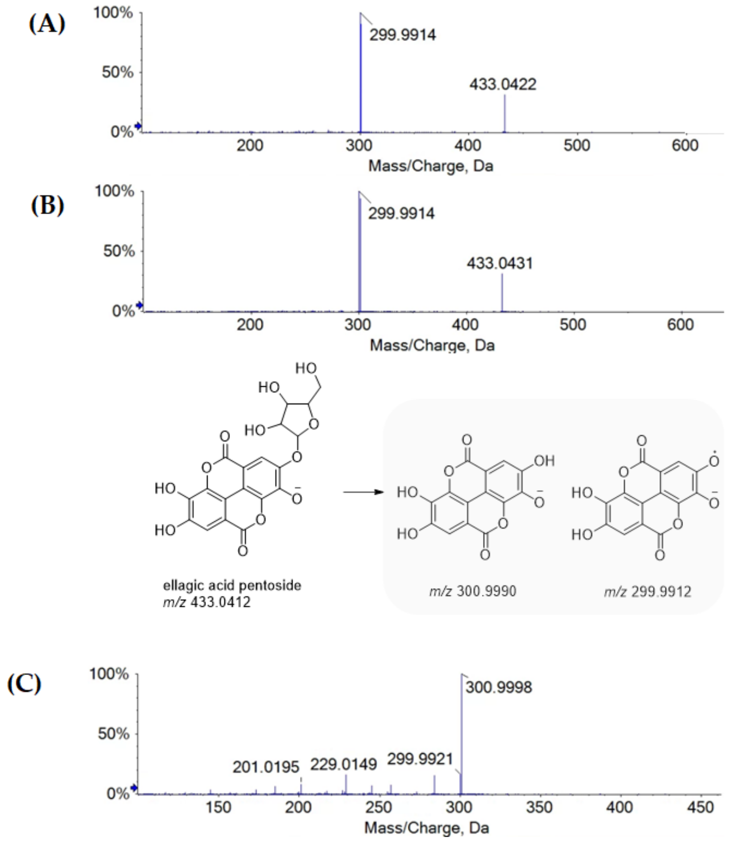

Figure S8. TOF-MS/MS spectra of compounds **27** (A) and **28** (B), likely ellagic acid pentoside isomers (whose fragmentation pattern is showed below), and of compound **29** (C).

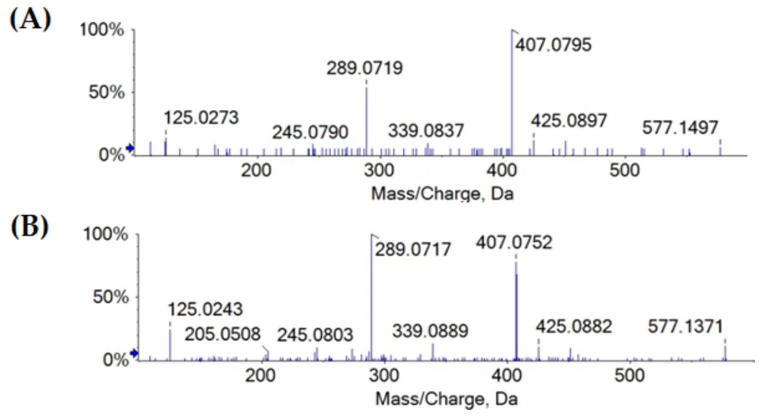

Figure S9. TOF-MS/MS spectra of B-type procyanidin isomers **16**(A) and **17** (B).

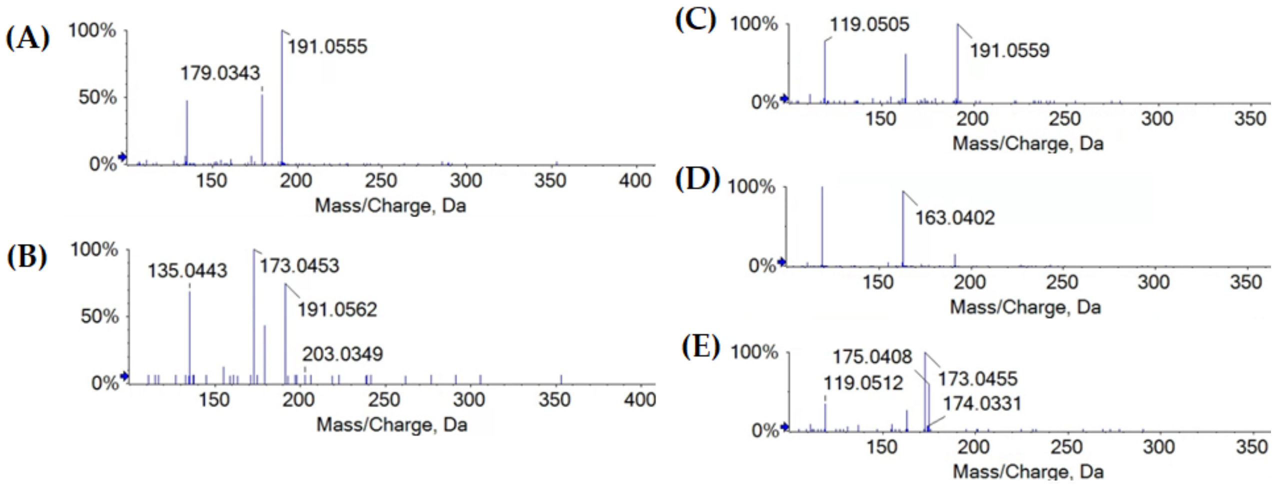

Figure S10. TOF-MS/MS spectra of chlorogenic acids: (A) 3-O-caffeoyl quinic acid (**11**); (B) 4-O-caffeoyl quinic acid (**19**); (C) 5-O-*p*-coumaroyl quinic acid (**14**); (B) 3-O-*p*-coumaroyl quinic acid (**19**); (E) 4-O-*p*-coumaroyl quinic acid (**21**).

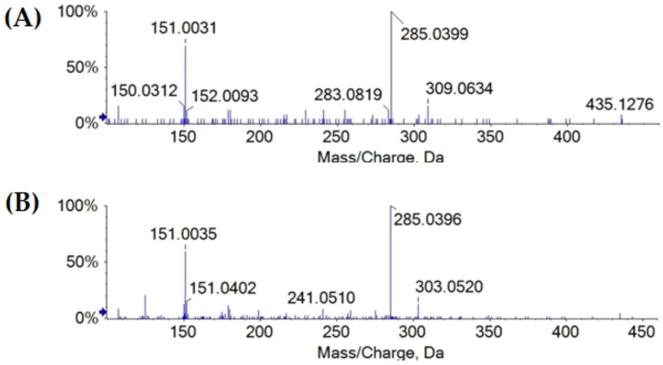

Figure S11. TOF-MS/MS spectra of taxifolin pentoside isomers: (A) **25**; (B) **26**.

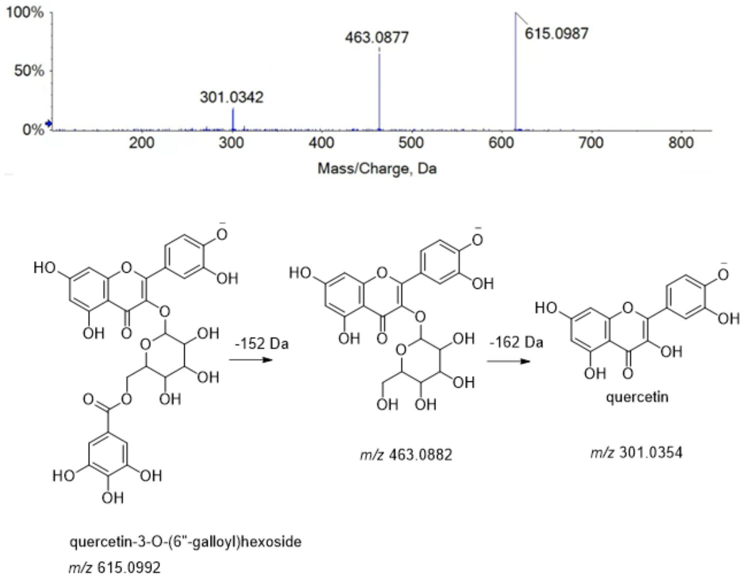

Figure S12. TOF-MS/MS spectra of quercetin glycoside **27**.

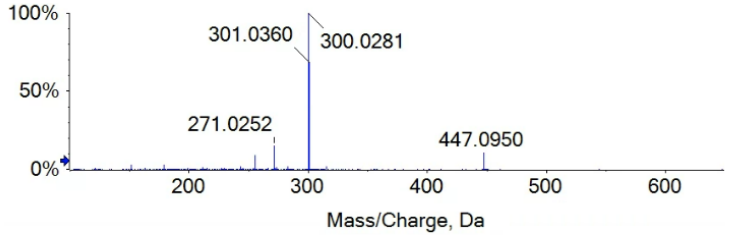

Figure S13. TOF-MS/MS spectra of quercetin glycoside **32**.
